# Dose–Concentration Relationship and Clinical Outcomes of Duloxetine in Generalized Anxiety Disorder

**DOI:** 10.3390/jcm15031211

**Published:** 2026-02-04

**Authors:** Ozgur Baykan, Sinan Altunoz, Dicle Yilmaz Uyanik, Hayriye Baykan

**Affiliations:** 1 Department of Medical Biochemistry, Faculty of Medicine, Balikesir University, Balikesir 10145, Turkey; ozgurbaykan@gmail.com; 2 Department of Mental Health and Diseases, Balikesir City Hospital, Balikesir 10100, Turkey; 3 Department of Mental Health and Diseases, Uskudar State Hospital, Istanbul 34662, Turkey; 4 Department of Mental Health and Diseases, Faculty of Medicine, Balikesir University, Balikesir 10145, Turkey

**Keywords:** duloxetine, anxiety disorders, plasma concentration, therapeutic drug monitoring

## Abstract

**Background/Objectives**: This study aimed to investigate plasma duloxetine concentrations, factors influencing these concentrations, and the relationship between plasma levels and clinical response in patients diagnosed with generalized anxiety disorder who were treated with duloxetine. Additionally, the study evaluated whether dose escalation resulted in proportional increases in plasma concentration and assessed the clinical utility of therapeutic drug monitoring (TDM) for duloxetine. **Methods**: In this study, plasma duloxetine levels were analyzed in 68 patients with generalized anxiety disorder who had been receiving duloxetine treatment for at least three months. A review of digital medical files revealed that duloxetine was initiated at 30 mg/day in all patients, and doses were increased to 60 or 90 mg/day in those with insufficient symptom improvement. Digital medical files indicated that participants had been on the final prescribed duloxetine dose for at least four weeks at the time of plasma level measurement. Baseline Hamilton Anxiety Rating Scale (HAM-A) and Generalized Anxiety Disorder-7 (GAD-7) scores were compared with scores obtained on the day of blood sampling. Plasma duloxetine concentrations were quantified using liquid chromatography–tandem mass spectrometry (LC-MS/MS). Associations between dose, plasma concentration, and clinical response were statistically analyzed. **Results**: Plasma duloxetine concentrations increased significantly following dose escalation. However, no significant correlation was observed between plasma concentrations and percentage change in HAM-A scores. Although patients receiving 60 or 90 mg/day had higher plasma levels than those maintained on 30 mg/day, clinical improvement did not differ significantly between dose groups. In addition to dose, increasing age and non-smoking status were associated with higher plasma duloxetine concentrations. **Conclusions**: Duloxetine demonstrates a predictable dose–concentration relationship; however, in this response-guided titration setting, plasma concentrations were not consistently associated with clinical improvement. Accordingly, these findings suggest that routine therapeutic drug monitoring may not consistently predict clinical response to duloxetine in generalized anxiety disorder; nevertheless, considering the study’s limitations, it could still offer clinically relevant insights in selected pharmacokinetically sensitive or treatment-resistant cases.

## 1. Introduction

Anxiety disorders are among the most prevalent psychiatric conditions worldwide and are associated with significant functional impairment, chronicity, and reduced quality of life [1]. Although pharmacological treatment with antidepressants, including SSRIs and SNRIs, constitutes a first-line approach [2], a substantial proportion of patients fail to achieve full remission, and treatment response remains highly variable [3].

Treatment response varies widely among individuals, and a substantial proportion of patients show only a partial response to treatment [4,5]. Therefore, understanding the biological, clinical, and pharmacokinetic factors influencing antidepressant response is crucial for the development of personalized treatment strategies [6].

Serotonin–norepinephrine reuptake inhibitors (SNRIs) are an important pharmacological option in depressive disorders due to their dual mechanisms, which enhance both serotonergic and noradrenergic neurotransmission. Duloxetine, one of the most commonly prescribed SNRIs, has been shown to be effective not only for depressive symptoms but also for comorbid anxiety- and pain-related syndromes [7]. Duloxetine generally exhibits linear pharmacokinetics; however, there is marked interindividual variability in plasma concentrations at the same dose, influenced by factors such as CYP1A2 and CYP2D6 metabolism, age, liver function, smoking, and concomitant medications [8,9]. In routine clinical practice, duloxetine treatment is typically initiated at a dose of 30 mg/day and increased to 60 mg/day when early response is inadequate [7].

Given this variability, therapeutic drug monitoring may help identify optimal plasma concentrations associated with clinical response. However, for antidepressants, evidence linking therapeutic drug monitoring to clinical efficacy is primarily available for older agents such as tricyclic antidepressants [10,11,12]. In contrast, the evidence for SSRIs and SNRIs remains inconclusive [13]. Previous studies investigating the concentration–response relationship for duloxetine have shown that therapeutic drug monitoring of duloxetine is useful for treatment optimization [14,15]. However, there are also studies indicating that higher plasma levels of duloxetine are not associated with greater clinical improvement [16]. These conflicting findings raise important clinical questions, including whether higher duloxetine doses are associated with higher plasma concentrations, whether elevated plasma concentrations lead to improved clinical outcomes, and whether measuring duloxetine blood levels can help clinicians distinguish pharmacokinetic failure from true treatment resistance.

Although duloxetine has been extensively studied in major depressive disorder, data on its pharmacokinetic–clinical relationships in anxiety disorders remain limited. Anxiety disorders may differ from depression in clinically relevant features, including autonomic arousal and noradrenergic–serotonergic balance, which could influence dose requirements and treatment response to serotonin–norepinephrine reuptake inhibitors. Moreover, interindividual variability in tolerability and dose escalation patterns may contribute to distinct concentration–response dynamics [17,18,19]. Therefore, findings from depressive populations may not be fully generalizable to anxiety disorders, supporting the need to evaluate duloxetine dose–concentration relationships and the clinical relevance of therapeutic drug monitoring specifically in this population.

Accordingly, our study investigated whether increasing the duloxetine dose from 30 mg/day to 60 or 90 mg/day resulted in a proportional increase in plasma concentrations and whether plasma duloxetine levels were associated with changes in anxiety severity. While it was assumed that dose escalation would increase plasma drug levels in a predictable manner, it was hypothesized that a significant relationship could be observed between plasma concentrations and clinical response. The overall aim of this study was to evaluate whether therapeutic drug monitoring provides a significant clinical benefit in guiding duloxetine treatment for generalized anxiety disorder.

## 2. Materials and Methods

The study included patients who attended the Psychiatry Outpatient Clinic of Balıkesir University Faculty of Medicine Hospital between 2021 and 2023 and had been using duloxetine for at least three months. The study protocol was approved by the Clinical Research Ethics Committee of Balıkesir University Faculty of Medicine (Decision No: 2020/39), and informed written consent was obtained from all participants. The research was supported by the Scientific Research Project Unit of Balıkesir University (2019/091).

The study has a retrospective, naturalistic, and observational design. Given the absence of a control group, the lack of prospective features, the impossibility of any intervention in the participants’ treatment processes due to the study design, the absence of randomization or blinding, and the lack of any influence of the study design on clinical decision-making, the study does not meet the definition of a clinical trial and therefore does not require pre-registration.

In this study, blood samples were collected to measure plasma duloxetine levels when patients receiving duloxetine treatment presented to the psychiatric outpatient clinic for their third-month follow-up visits. All other data were obtained retrospectively through a review of the patients’ medical records. Participants were selected from patients who had been diagnosed with generalized anxiety disorder according to DSM-5 criteria by a psychiatrist and who had been receiving duloxetine treatment for at least 12 weeks. Patients whose diagnoses were confirmed by a structured clinical interview (SCID-5) conducted by a psychiatrist were included in the study. Patients with comorbid syndromic depressive disorders and those receiving psychotherapy were not included in the study.

Pre-treatment Hamilton Anxiety Rating Scale (HAM-A) and Generalized Anxiety Disorder-7 (GAD-7) scores were obtained from the patients’ digital medical files. Review of these files indicated that all patients were initiated on duloxetine at a dose of 30 mg/day, with subsequent dose increases to 60 mg/day or 90 mg/day in cases of insufficient clinical improvement, typically within the first month of treatment. At the time of plasma duloxetine measurement, review of the participants’ digital medical files indicated that they had been maintained on the final prescribed duloxetine dose for at least four weeks.

Individuals with bipolar disorder, psychotic disorders, active substance or alcohol use disorder, a history of serious neurological disease (e.g., epilepsy or dementia), hepatic or renal insufficiency, or who were pregnant or breastfeeding were excluded from the study. In addition, patients using CYP1A2 inhibitors (e.g., fluvoxamine) concomitantly with duloxetine were excluded because of their potential effects on plasma drug concentrations. Because study inclusion required ongoing duloxetine treatment for at least 12 weeks, patients who discontinued treatment early due to lack of response or poor tolerability were excluded from the study sample. Data on patients who initiated duloxetine but discontinued before 12 weeks were not systematically available and could not be reliably reconstructed from the records. Participants were divided into three groups according to their duloxetine dosage during treatment: 30 mg/day, 60 mg/day, and 90 mg/day. Given the response-guided titration strategy, baseline severity was not used to define comparable dose groups, and between-group comparisons were interpreted descriptively.

### 2.1. Data Collection and Clinical Measures

Participants’ sociodemographic and clinical characteristics (age, sex, duration of illness, number of previous episodes, BMI, etc.) were recorded using a semi-structured form. Anxiety severity was assessed using the Hamilton Anxiety Rating Scale (HAM-A) and Generalized Anxiety Disorder–7 (GAD-7) scale [20,21]. Clinical response was defined as a reduction of at least 50% from the baseline HAM-A score [22]. Clinical change was operationalized as the percentage change in raw HAM-A and GAD-7 scores between baseline and the assessment performed on the day of blood sampling.

### 2.2. Plasma Duloxetine Level Measurement

To determine plasma duloxetine levels, blood samples were collected from patients in the morning after 8–12 h of fasting and prior to the daily duloxetine dose, in order to approximate trough plasma concentrations. Duloxetine was prescribed as a once-daily medication. Medication adherence was assessed based on patient self-reporting and a review of digital medical records, and patients reporting missed doses within the 24 h preceding blood sampling were not included in the analysis. Following centrifugation at 1300× *g*, 2 mL of plasma was transferred into Eppendorf tubes for analysis. Plasma samples were stored at −40 °C until analysis.

A liquid chromatography–tandem mass spectrometry (LC-MS/MS) method was developed and validated for the quantification of duloxetine in human plasma. Following protein precipitation with acetonitrile, chromatographic separation of duloxetine (TRC Reference Standards, Toronto, ON, Canada) and duloxetine-d7 maleate (stable isotope-labeled internal standard; TRC Reference Standards, Toronto, ON, Canada) was achieved using a C18 column (2.1 × 50 mm, 2.2 μm; Thermo Scientific, Waltham, MA, USA). Detection was performed using a Quantum Access Max LC-MS/MS mass spectrometer (Thermo Scientific, USA). Ionization was carried out by electrospray ionization (ESI) operated in positive ion mode. The mass transitions monitored were *m*/*z* 298.1 → 154.1 for duloxetine and *m*/*z* 305.2 → 154.1 for duloxetine-d7 maleate. The method was validated over a linear concentration range of 2.5–500 ng/mL. The limit of detection (LOD) and limit of quantification (LOQ) for duloxetine were approximately 0.6 ng/mL and 2.5 ng/mL, respectively, as determined based on signal-to-noise ratios of approximately 3:1 and 15:1. Chromatographic separation was performed under isocratic conditions using a mobile phase consisting of acetonitrile/water containing 0.1% formic acid, with a total run time of 4 min. The retention times for duloxetine and duloxetine-d7 maleate were both approximately 3.16 min.

### 2.3. Statistical Analysis

Statistical analyses were performed using SPSS version 25.0 (IBM Corp., Armonk, NY, USA). Descriptive statistics are presented as mean ± standard deviation, median (25th–75th percentiles), and minimum–maximum values. For comparisons between two independent groups, the independent sample *t*-test was used for normally distributed variables, whereas the Mann–Whitney U test was applied for non-normally distributed variables. Comparisons among more than two independent groups were conducted using analysis of variance (ANOVA) or the Kruskal–Wallis test, depending on data distribution. Categorical variables were evaluated using cross-tabulations and the chi-square test. The relationship between plasma duloxetine levels and anxiety scores was assessed using Spearman correlation analysis, taking data distribution into account. Baseline and 3-month values of the HAM-A and GAD-7 scales were compared using the paired samples *t*-test. Linear regression analysis was performed to identify variables affecting plasma duloxetine concentrations, with age, sex, BMI, and smoking status included as independent variables. A *p* value of <0.05 was considered statistically significant.

## 3. Results

### 3.1. Participant Characteristics

A total of 68 patients were included in the study, comprising 63 females and 5 males. The mean age of the participants was 48.9 ± 10.6 years. Of the patients, 21 were receiving duloxetine at a dose of 30 mg/day, 39 at 60 mg/day, and 8 at 90 mg/day. The mean ± SD BMI values for the 30 mg, 60 mg, and 90 mg groups were 32.4 ± 6.3, 30.6 ± 5.0, and 30.3 ± 6.2, respectively, with no statistically significant difference between the groups (*p* = 0.466). Forty-one participants (60.3%) were experiencing a first episode, while 27 (39.7%) had a history of two or more previous episodes.

### 3.2. Plasma Duloxetine Levels

**Figure 1:**
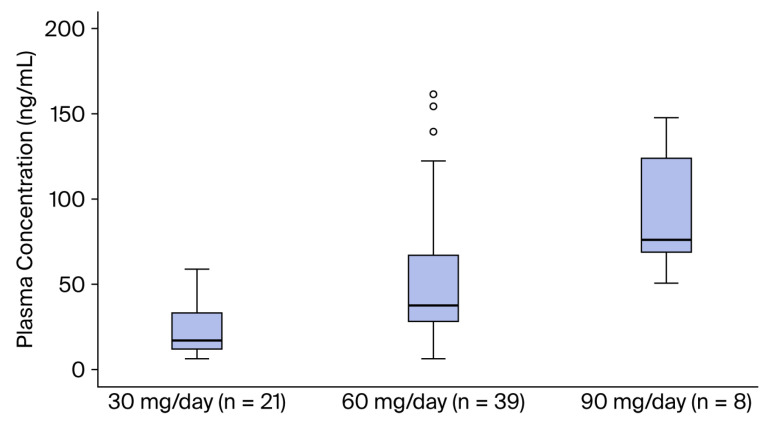
Plasma duloxetine concentrations (ng/mL) according to daily dose: 30 mg/day (*n* = 21), 60 mg/day (*n* = 39), and 90 mg/day (*n* = 8). Box plots show the median and interquartile range (25th–75th percentiles); whiskers indicate minimum and maximum values, and circles denote outliers.

The median (25th–75th percentile) plasma duloxetine concentration was 16.1 (11.2–36.0) ng/mL in patients receiving 30 mg/day, 36.7 (25.6–68.6) ng/mL in those receiving 60 mg/day, and 75.0 (67.5–132.3) ng/mL in patients receiving 90 mg/day. A statistically significant difference was observed among the groups (*p* < 0.001) (Table 1, Figure 1).

Pairwise comparisons were performed to determine between which groups the differences in plasma levels occurred. Statistically significant differences in plasma duloxetine concentrations were observed between the 30 and 60 mg/day groups (*p* = 0.006), the 30 and 90 mg/day groups (*p* < 0.001), and the 60 and 90 mg/day groups (*p* = 0.031).

**Figure 2:**
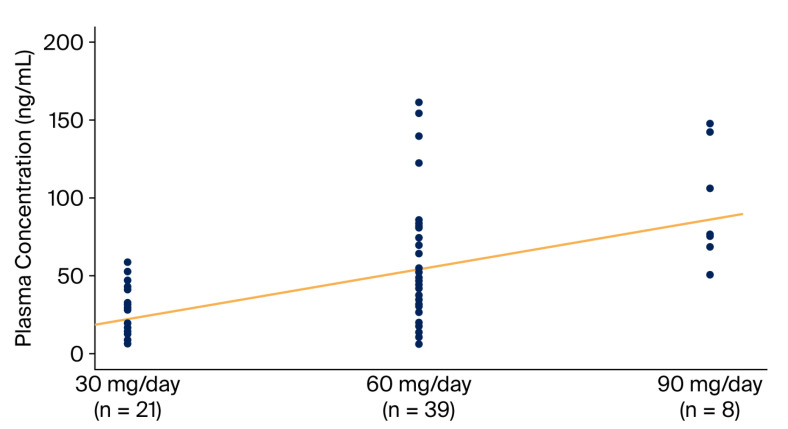
Relationship between daily duloxetine dose and plasma duloxetine concentration (ng/mL). Individual data points represent patients receiving 30 mg/day (*n* = 21), 60 mg/day (*n* = 39), and 90 mg/day (*n* = 8). The solid line indicates the fitted linear trend.

When the relationship between the prescribed dose and plasma concentrations was evaluated among participants receiving three different doses of duloxetine, a highly significant correlation was observed (r_s_ = 0.559, *p* < 0.001) (Figure 2).

To determine the effects of variables on plasma duloxetine concentration, a multiple linear regression analysis was performed. Accordingly, dose (β = 0.602, *p* < 0.001), age (β = 0.237, *p* = 0.024), and non-smoking status (β = 0.218, *p* = 0.042) were found to have a positive and statistically significant effect on plasma concentration, whereas BMI did not have a significant effect (*p* = 0.469) (Table 2). A one-unit increase in age, with other variables held constant, was associated with a positive increase of 0.857 units in plasma concentration (95% CI: 0.119–1.594). Similarly, plasma concentrations were on average 17.24 units higher in non-smokers compared with smokers (95% CI: 0.643–33.841). A one-unit increase in dose resulted in a 1.227-unit increase in plasma concentration (95% CI: 0.822–1.632). According to the standardized coefficients, daily dose contributed the most to the explanatory power of the model (β = 0.602).

### 3.3. Clinical Change

At the third month of treatment, HAM-A and GAD-7 scores assessed at the time of blood sampling for plasma level determination were compared with the scores obtained on the day duloxetine treatment was initiated. After three months of treatment, HAM-A scores were found to be significantly reduced compared with the baseline in patients receiving 30 mg/day (*p* < 0.001), 60 mg/day (*p* < 0.001), and 90 mg/day (*p* < 0.001). Similarly, GAD-7 scores at the end of three months were significantly lower than baseline in patients treated with 30 mg/day (*p* < 0.001), 60 mg/day (*p* < 0.001), and 90 mg/day (*p* < 0.001).

Percentage changes in HAM-A and GAD-7 scores were calculated by dividing the difference between baseline and 3-month scores by the baseline score and multiplying by 100. The mean ± SD percentage change in HAM-A scores was 84.3 ± 11.5% in patients receiving 30 mg/day, 85.1 ± 10.1% in those receiving 60 mg/day, and 86.0 ± 10.7% in those receiving 90 mg/day, with no statistically significant difference between the groups (*p* = 0.923). The mean ± SD percentage change in GAD-7 scores was 84.6 ± 15.6% in the 30 mg/day group, 84.5 ± 11.7% in the 60 mg/day group, and 88.3 ± 13.1% in the 90 mg/day group, and no statistically significant difference was observed among the groups (*p* = 0.750). It should be noted that all patients initially started treatment at a dose of 30 mg/day, and dose escalation was implemented at the first-month follow-up in patients who did not demonstrate sufficient clinical improvement. Consequently, the study sample represents a clinically selected group of patients who tolerated and responded to duloxetine treatment, which should be considered when interpreting the magnitude of symptom improvement.

## 4. Discussion

In this study, plasma duloxetine concentrations, variables potentially affecting plasma levels, and their association with clinical response were investigated in patients diagnosed with generalized anxiety disorder who had been receiving duloxetine treatment for at least three months. Plasma duloxetine concentrations were found to increase proportionally with dose escalation, confirming the linear pharmacokinetics of duloxetine, consistent with previous studies. Despite the dose–concentration relationship, no significant correlation was observed between plasma duloxetine levels and the percentage reduction in HAM-A and GAD-7 scores. Importantly, under a response-guided titration framework characterized by confounding by indication, similar levels of symptom improvement across dose groups should not be interpreted as evidence that dose has no clinical effect, but rather as a reflection of substantial interindividual variability in dose requirements.

Previous pharmacokinetic studies have demonstrated that duloxetine exhibits linear, dose-proportional increases in plasma concentration within the therapeutic dose range [14,23]. However, a recent meta-analysis reported that in some studies, no significant correlation was found between mean doses and mean plasma levels when linear regression analysis was used [24]. In our study, patients receiving 60 and 90 mg/day had significantly higher plasma duloxetine concentrations compared with those who continued treatment at 30 mg/day. These findings support existing evidence that, despite known interindividual differences related to CYP2D6 and CYP1A2 activity, the absorption and metabolism of duloxetine are generally predictable.

Most studies examining the concentration–clinical response relationship for duloxetine have been conducted in patients with major depressive disorder and have yielded complex and mixed findings. While some studies have demonstrated a significant correlation between duloxetine plasma levels and clinical response in patients with major depressive disorder [14,15], another study in geriatric patients reported that higher duloxetine plasma concentrations were not associated with greater clinical improvement and might even adversely affect clinical outcomes [16].

To the extent available in the literature, no study directly examining the relationship between duloxetine plasma levels and clinical improvement in patients followed up with anxiety disorders has been found. However, another follow-up study in patients diagnosed with major depressive disorder found a significant relationship between duloxetine plasma levels and clinical improvement in terms of anxiety scores (HAM-A scores) [25]. Therefore, our study is of particular importance in that it investigates the relationship between duloxetine plasma levels and clinical response specifically in patients with generalized anxiety disorder.

When considering why plasma concentrations do not correlate with clinical improvement, anxiety disorders are heterogeneous in terms of clinical presentation, symptom course, and treatment response, and plasma drug levels may not adequately capture pharmacodynamic variability such as receptor sensitivity, differences in neurotransmitter systems, or downstream neuroadaptive processes. In addition, clinical response to SNRIs may depend more on transporter occupancy thresholds than on plasma concentrations alone. A positron emission tomography (PET) study evaluating the relationship between duloxetine plasma levels and serotonin and norepinephrine transporter occupancy rates showed that plasma concentrations above 10–15 ng/mL were associated with approximately 80% serotonin transporter (SERT) occupancy [26], while another study showed that plasma concentrations above 58 ng/mL are required to achieve about 50% norepinephrine transporter (NET) occupancy [27]. These findings suggest that relatively low duloxetine plasma levels may be sufficient to achieve adequate SERT occupancy and therapeutic response in some patients, whereas others may require higher plasma concentrations to reach sufficient NET occupancy. Unmeasured factors such as genetic variability in drug metabolism, differences in receptor sensitivity, or clinical subtypes of anxiety disorders may further contribute to this heterogeneity in dose requirements. Consistent with this interpretation, although all patients in our study were initiated on duloxetine at a dose of 30 mg/day, this dose was sufficient in 30.8% of patients, while the remaining patients required dose escalation. This pattern supports the notion that duloxetine dose requirements vary considerably between individuals rather than indicating an absence of dose-related effects.

Previous studies investigating the effect of age on duloxetine plasma concentrations did not report a significant association [15,16]. In contrast, our findings demonstrate a significant positive relationship between increasing age and higher plasma duloxetine levels. This can be attributed to age-related pharmacokinetic changes, including reduced hepatic blood flow and diminished cytochrome P450 enzyme activity—particularly CYP1A2 and CYP2D6—which play a major role in duloxetine metabolism. Such physiological changes may lead to reduced clearance and increased systemic exposure in older individuals.

Clinically, this age-related increase in plasma concentrations did not translate into greater symptom improvement in our sample, suggesting that higher drug exposure alone may not enhance therapeutic outcomes in anxiety disorders. Although adverse effects were not systematically evaluated in this study, these findings underscore the importance of cautious dose titration in older patients, as higher plasma concentrations may have clinical relevance primarily from a tolerability perspective rather than in terms of additional symptomatic benefit.

Smoking increases the metabolism of drugs that are substrates of cytochrome P450 (CYP) 1A2 due to CYP induction. Duloxetine, an antidepressant that acts as a serotonin–norepinephrine reuptake inhibitor, is primarily metabolized via the CYP1A2 pathway. Studies evaluating duloxetine plasma levels in smokers and non-smokers have shown that duloxetine blood concentrations are lower in smokers than in non-smokers [9,28]. In line with these reports, smoking status was also identified as a significant determinant of plasma duloxetine exposure in the present study. From a clinical perspective, these findings support the potential role of plasma duloxetine measurement may be informative in selected situations characterized by increased pharmacokinetic variability, rather than as a routine monitoring strategy. Such situations may include suspected non-adherence, unexpectedly low plasma levels despite adequate dosing, pronounced interindividual variability related to smoking or age, potential CYP1A2 or CYP2D6 genetic polymorphisms, hepatic impairment, or complex polypharmacy involving CYP inhibitors or inducers. Importantly, while our data support the relevance of smoking as a pharmacokinetic modifier, the present study was not designed to define therapeutic thresholds or to establish concentration–adverse effect relationships, and such applications of TDM should be examined in prospective studies with systematic assessment of tolerability and adherence.

This study has several limitations. As a retrospective, naturalistic observational study, it does not allow causal conclusions to be drawn about concentration–response relationships. A major limitation is the presence of substantial selection bias. Because study inclusion required continued duloxetine treatment for at least 12 weeks, patients who discontinued treatment early due to insufficient response or poor tolerability were excluded, resulting in a clinically selected, response-enriched sample. This selection process likely contributed to the unusually large symptom improvements observed across all dose groups and limits the interpretability of between-group comparisons as well as the assessment of concentration–effect relationships. Moreover, because dose escalation was response-guided, patients receiving 60/90 mg/day likely represented those with poorer early response and/or higher baseline symptom burden compared with those maintained on 30 mg/day, further complicating direct comparisons. HAM-A and GAD-7 scores were available only at baseline and at the time of blood sampling, precluding detailed analyses of symptom trajectories. In addition, CYP2D6 and CYP1A2 genotyping was not performed, which may have limited insight into interindividual variability in duloxetine metabolism. Finally, although the sample size was larger than that of many previous duloxetine concentration studies, it restricted robust subgroup and sensitivity analyses. Future prospective studies with repeated assessments, larger samples, and genetic stratification are needed to clarify concentration–response relationships and the potential role of therapeutic drug monitoring in anxiety disorders.

Despite these limitations, this study adds novel data to a relatively underexplored area by characterizing duloxetine plasma concentrations in patients with generalized anxiety disorder. We observed a clear dose–concentration relationship; however, in this response-guided titration setting and clinically selected sample, plasma levels were not reliably associated with the magnitude of symptom improvement. This finding should be interpreted cautiously, as the study design and selection of patients who tolerated and continued treatment may have limited the ability to detect a true concentration–response relationship. Accordingly, rather than indicating a lack of clinical relevance of dose or drug exposure, these results suggest that the predictive value of routine therapeutic drug monitoring for clinical response cannot be reliably determined in this context. Nevertheless, TDM may still be informative in selected pharmacokinetically sensitive or treatment-resistant cases, particularly when adherence, drug interactions, or metabolic variability are of concern. Age and smoking status emerged as significant determinants of plasma exposure, underscoring the relevance of individual pharmacokinetic factors in clinical practice. Future prospective studies with larger samples, inclusion of early non-responders, genetic stratification, and longitudinal symptom trajectories are warranted to better identify patient subgroups in whom TDM-guided duloxetine dosing may be clinically beneficial.

## Figures and Tables

**Table 1:** Plasma duloxetine concentrations according to daily duloxetine dose.

Oral Duloxetine Dose	Plasma Duloxetine Concentrations Median (25th–75th Percentile), ng/mL	*p* Value
30 mg/day (*n* = 21)	16.1 (11.2–36.0)	<0.001
60 mg/day (*n* = 39)	36.7 (25.6–68.6)	
90 mg/day (*n* = 8)	75.0 (67.5–132.3)	

**Table 2:** Linear regression analysis of factors affecting plasma duloxetine concentration.

	Unstandardized Coefficients		Standardized Coefficients	t	Sig.	95.0% Confidence Interval for B	
	B	Std. Error	Beta			Lower Bound	Upper Bound
Age	0.857	0.369	0.237	2.321	0.024	0.119	1.594
BMI	0.496	0.680	0.071	0.729	0.469	−0.863	1.855
Non-smoking	17.242	8.306	0.218	2.076	0.042	0.643	33.841
Dose	1.227	0.203	0.602	6.052	<0.001	0.822	1.632

## Data Availability

The data that support the findings of this study are available from the corresponding author upon reasonable request.

## Abbreviations

The following abbreviations are used in this manuscript:

HAM-A	Hamilton Anxiety Rating Scale
GAD-7	Generalized Anxiety Disorder-7 Scale
SSRI	Selective Serotonin Reuptake Inhibitor
SNRI	Serotonin–Norepinephrine Reuptake Inhibitor
CYP	Cytochrome P450
BMI	Body Mass Index
LC-MS/MS	Liquid chromatography–tandem mass spectrometry
HPLC	High-Performance Liquid Chromatography
SD	Standard Deviation
CI	Confidence Interval
r_s_	Spearman’s rank correlation coefficient
SERT	Serotonin transporter
NET	Norepinephrine transporter
PET	Positron emission tomography
